# Esthetic Rehabilitation of Anterior Dentition with Different Types of Ceramic Restorations: Two Case Reports

**DOI:** 10.1055/s-0041-1732805

**Published:** 2021-10-01

**Authors:** Michal Krump, Zelmira Krumpova

**Affiliations:** 1 Department of Esthetic Dentistry, Private Dental Practice, Puchov, Slovak Republic

**Keywords:** esthetic rehabilitation, ceramic veneers, all-ceramic full crowns, lithium disilicate

## Abstract

All-ceramic systems represent an excellent restorative alternative for fixed dental prostheses, single crowns, and veneers in the anterior dentition. With respect to improved mechanical properties, lithium disilicate ceramic material provide a broad range of indications, and extended veneers can serve as an alternative to full crowns. Although ceramic veneers represent a more conservative approach compared to crowns, the correct indication is essential to achieving the ideal outcome. The following case reports describe two types of fixed restorations of the anterior dentition: extended lithium disilicate ceramic veneers and lithium disilicate full crowns. Factors influencing treatment selection for each type of restorations are presented.

## Introduction

All-ceramic systems represent an excellent restorative alternative for fixed dental prostheses, single crowns, and veneers in the anterior dentition. 1 2

Full-coverage crowns offer predictable treatment options, but a certain amount of tooth material must be removed to allow space for the required thickness of the restorative material. Progress in adhesive technologies and in ceramic materials strength has made possible a more conservative restoration techniques, such as thin ceramic veneers. 3 4 5

Silica-based all-ceramics have been proven effective in numerous long-term clinical studies as an appropriate material for esthetic restorations. 6 7 8

Feldspathic ceramics provide the best representation of the optical properties of the natural tooth. Because their mechanical properties are low (flexural strength of 60–70 MPa), these materials are generally used for veneering to metal or ceramic substructures or for veneer fabrication by using the platinum foil or refractory die technique. 9

The group of ceramic materials that contains a glass matrix with varying amounts of crystalline mineral including leucite, lithium disilicate, or fluoroapatite.

These filler particles grow inside the glass matrix during the crystallization process to improve mechanical properties and to control optical effects such as color, opalescence, translucency, and opacity. 9 10 11

Fluoroapatite-based ceramic materials consist of fluoroapatite crystals in an aluminosilicate glass matrix are used to veneer porcelain substrates for developing the final morphology and shade of the restoration. 12 13

The leucite glass-ceramic material consists of a glass matrix surrounding leucite crystals. In concentrations of 35% to 55%, the leucite is used as a reinforcing material. Leucite possesses an index of refraction similar to that of feldspathic glasses, which allows the translucency to be maintained. 9 11 .

Lithium disilicate ceramic material consists of a glass matrix highly filled with lithium silicate, with micron-size lithium disilicate crystals in between. 10 14

These incorporated crystals significantly increase the materials strength (400 MPa), and despite a high crystalline content, the low refractive index of the lithium disilicate crystals allows the material to maintain a high translucency. 9 15 16

With respect to this, lithium disilicate ceramic material allow the production of thin ceramic veneers with minimum reduction of dental tissues. 4 5

In the cases of extended defect-oriented preparation designs, lithium disilicate ceramics veneers offer an alternative to full crowns in the anterior dentition. 17 18

Although ceramic veneers represent a more conservative approach compared to crowns, the correct indication is essential to achieving the ideal outcome in terms of longevity. 19 20

The following case reports describe the esthetic rehabilitation of anterior dentition involving both type of restorations. The lithium disilicate ceramic material was used in both cases because of its mechanical properties, high esthetics, and abrasion compatibility with the opposing natural dentition. 21

## Case Reports

### Ceramic Veneers

**Fig. 1:**
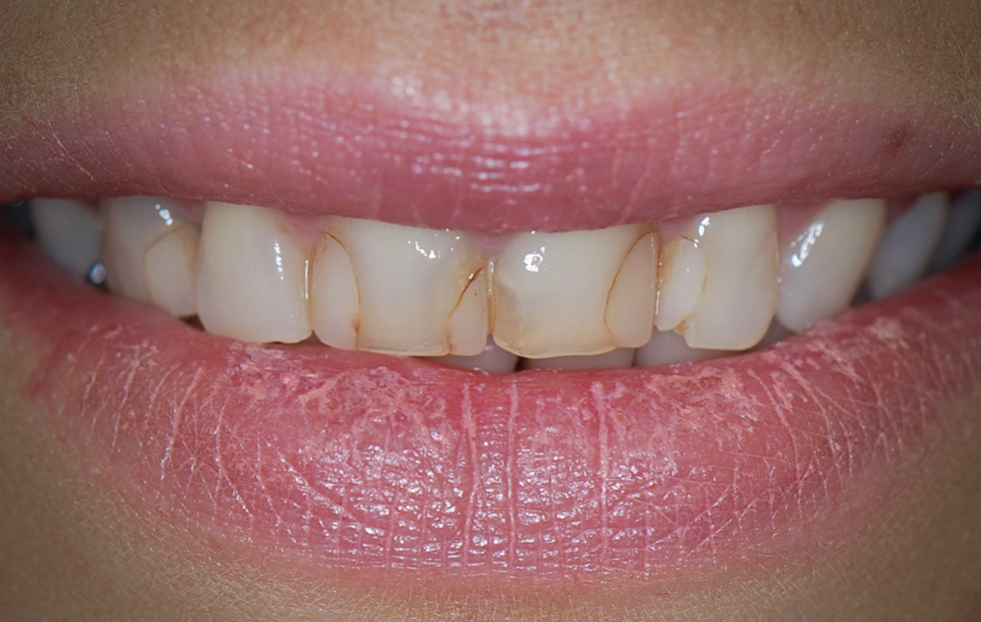
Preoperative labial view. Disharmony between the upper incisors with insufficient length of the central incisors results in inverted incisal edge configuration.

**Fig. 2:**
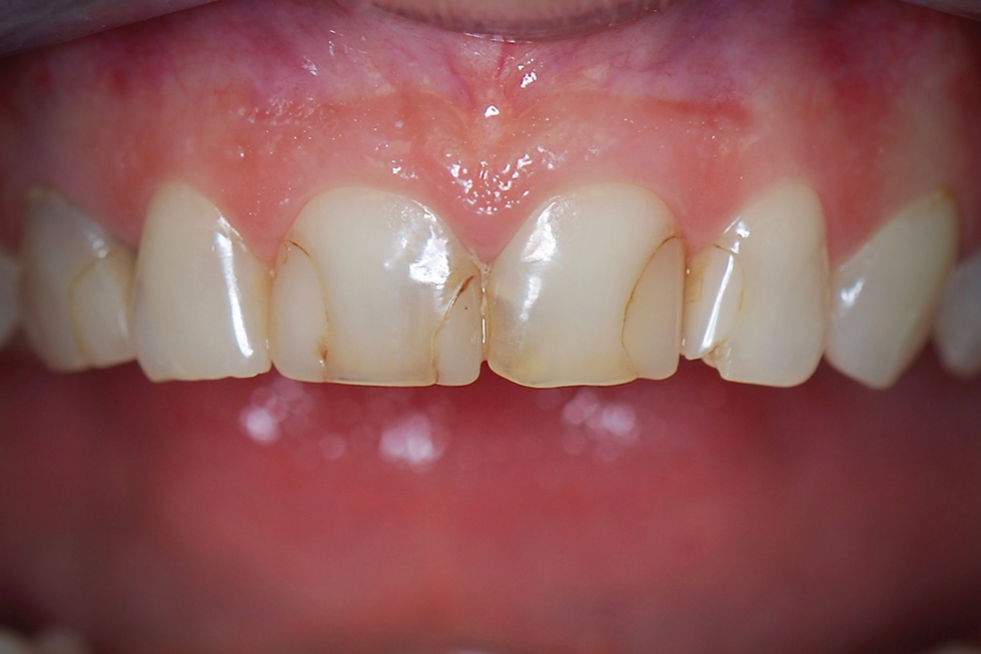
Preoperative intraoral view. Note the apparent unesthetic appearance of the anterior dentition with unsatisfactory composite resin fillings.

The 30-year-old female patient presented herself for esthetic rehabilitation of the anterior teeth. The patient’s chief complaint was unesthetic appearance of the upper dentition. First, the extra-oral clinical examination was done. Subsequently, the intra-oral examination revealed Angle Class I occlusion right side and half Angle Class II malocclusion left side, presence of unsatisfactory Class III and IV composite resin fillings with no carious lesions (marginal discolorations probably caused by inadequate etching or bonding around the preparation margins as well as composite overhangs caused by inadequate finishing and polishing) and length disharmony between the central and lateral incisors. Periodontal evaluation found no pathologic probing depths. Tooth number 13 was endodontically treated with minimal discoloration at the cervical area. Radiographic examination revealed extended composite restorations; no carious lesions and endodontically treated tooth number 13 with no findings of periapical pathology. The quality of endodontic treatment was assessed with a favorable outcome. Evaluation of the patient’s medical history was insignificant. Based on examination (age, enamel thickness, no dentin exposure, no attrition of the palatal surfaces, and possibility to place the preparation margins on enamel) extended ceramic veneers were planned to restore the teeth number 13, 12, 11, 21, and 22. Because of possible value mismatch caused by different ceramic thickness, the extended ceramic veneer was used also for the restoration of the endodontically treated tooth number 13. Digital photography was performed to provide diagnostic information to the restorative team, such as visualization and quantification of a patient’s smile ( Fig. 1 and Fig. 2 ). The color shade was selected by using a IPS e.max Shade Guide (Ivoclar Vivadent).

The first phase of the veneer preparation involved the use of round-end diamond bur with 1.0 mm diameter (Edenta AG) to create three facial reduction grooves. The grooves were created by respecting the axial inclinations of the tooth and were subsequently evened by using the cylindric diamond bur with larger diameter (Edenta AG). The gingival margin was placed at the level of the gingival crest. The minimum reductions in tooth structure during preparation were as follows: cervical reduction was 0.3 mm, facial reduction was 0.3 to 0.5 mm, and incisal reduction with butt joint design was 1.0 mm. The veneer preparation and depth of reduction was controlled by using the silicon index.

**Fig. 3:**
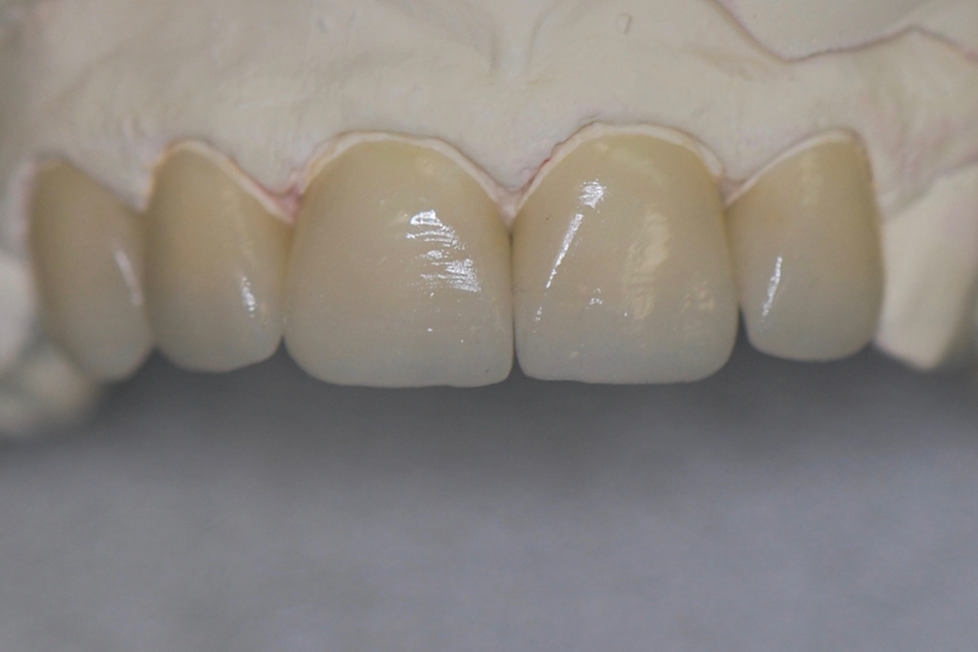
Labial view of lithium disilicate ceramic veneers.

**Fig. 4:**
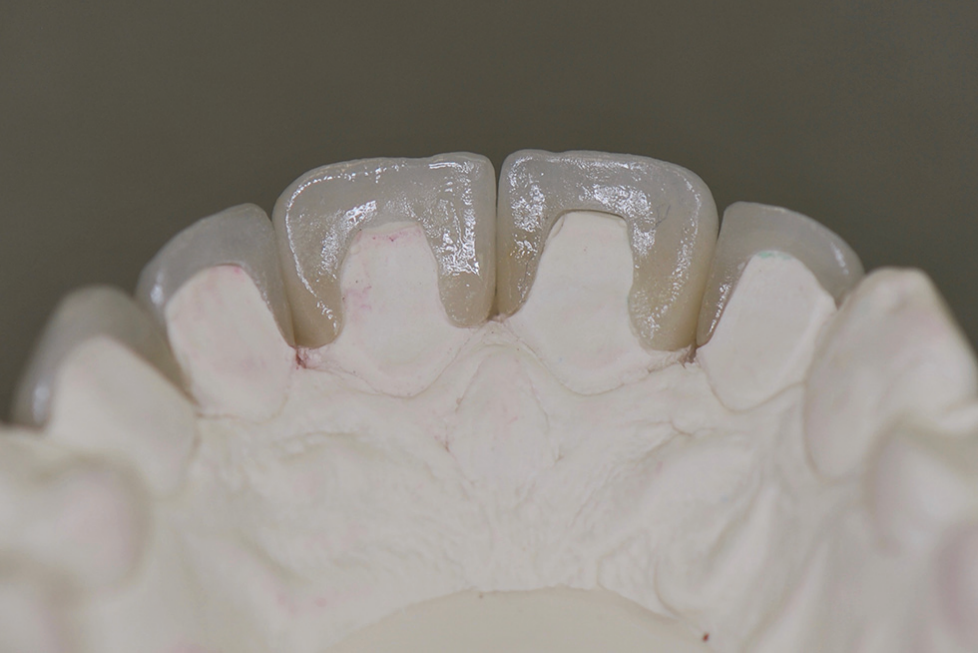
Palatal view of lithium disilicate ceramic veneers. Note the preparation based on the extension of the pre-existing composite resin fillings and preparation margins located exclusively in enamel.

The next phase consisted of the interproximal preparation with extended defect-oriented preparation design. If resin restorations were located at the preparation margins, the preparation was extended deeper into the palatal surfaces until the margins were on sound enamel. Extra-fine finishing diamonds were subsequently used to obtain smooth contours (Edenta AG). The next phase involved the impression at the same appointment, using addition silicone (Variotime, Kulzer) and a double-cord technique for gingival deflection (7 Siltrax AS, Pascal and 1 Ultrapak, Ultradent). Provisional restoration was created chairside with self-curing acrylic resin-based provisional restoration material (Structure 2SC, Voco). In the laboratory, the lithium disilicate ceramic (IPS e.max Press A1 HT, Ivoclar Vivadent) was used for fabrication of the veneers ( Fig. 3 and 4 ). The ingot was hot pressed at 915°C to flow viscously into the dental mold made by the lost wax technique to form the restorations and was held at this temperature for 15 minutes. The fully contoured restorations were further characterized with stains and glazed.

After 7 days (the time between the final impression and cementation), the provisional restoration was removed and luting procedures were initially performed by using a try-in test paste to select the best shade option (Variolink Esthetic Try-In-Paste, Ivoclar Vivadent). The interior surfaces of the veneers were etched with 9% buffered hydrofluoric acid (Porcelain Etch, Ultradent Products, Inc.) for 20 seconds to create surface roughness, followed by rinsing and air-drying. 22

**Fig. 5:**
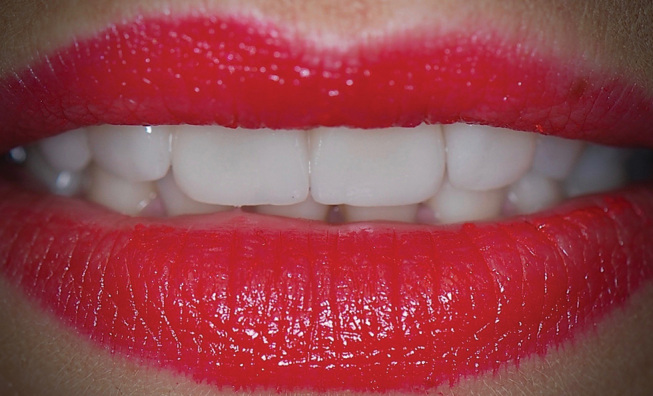
Final result 1 month after cementation. Note the harmonious association of incisal edges of upper anterior teeth with the lower lip during moderate smiling.

**Fig. 6:**
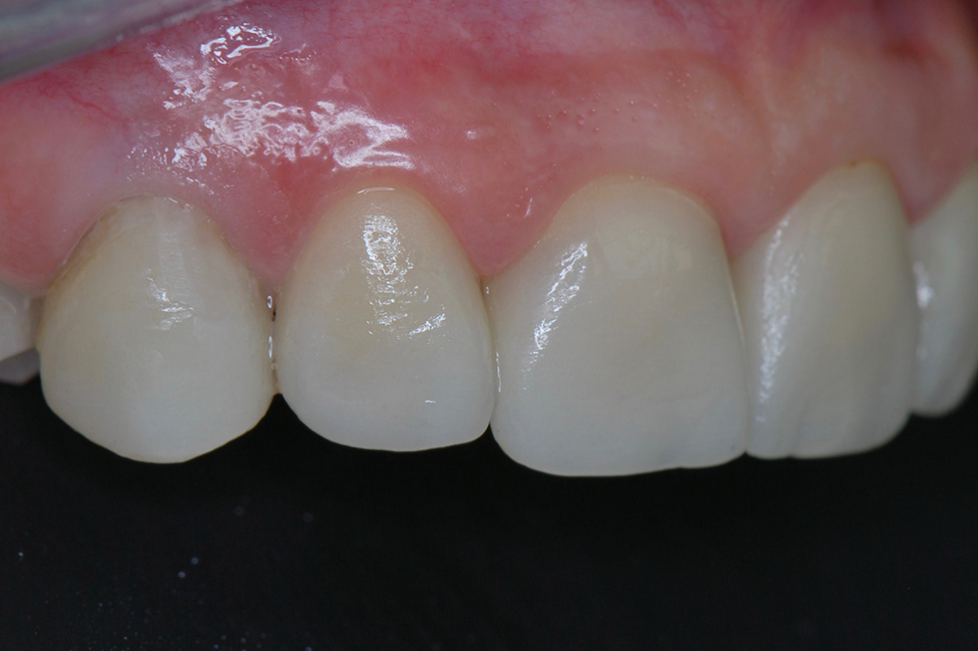
Intraoral view of right side. 18-month follow up of the ceramic veneers showing a favourable periodontal situation.

**Fig. 7:**
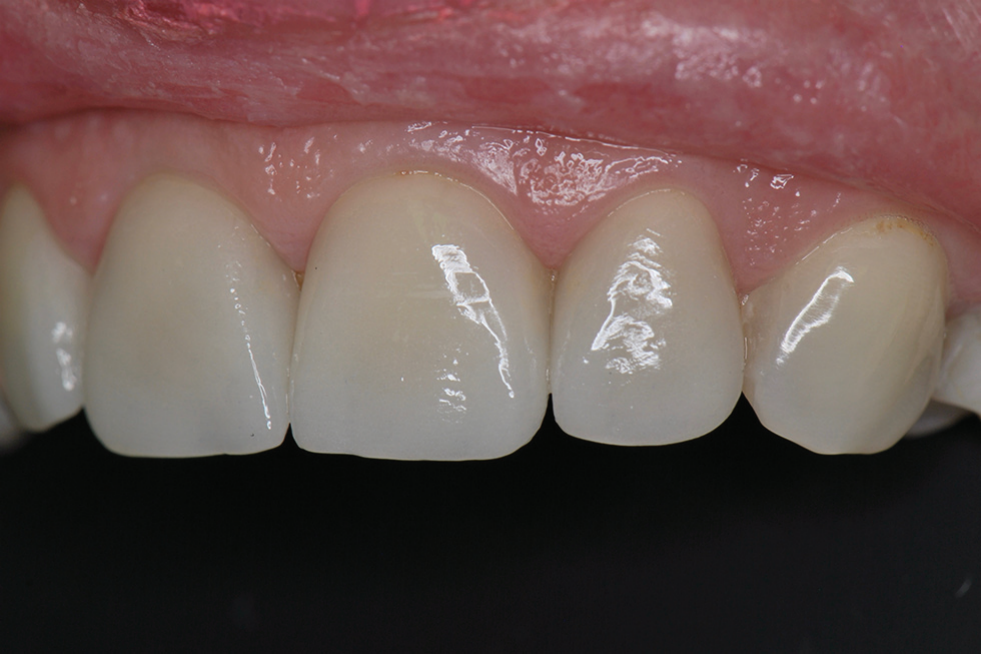
Intraoral view of left side. 18-month follow up of the ceramic veneers showing a favourable periodontal situation.

A silane agent was then applied to the etched ceramic surface of the veneers and air-dried (Monobond plus, Ivoclar Vivadent). The gingival displacement was acquired by using a retraction cord (7 Siltrax AS, Pascal). Subsequently, adequate surface treatment for the dental tissues was done. The teeth were cleaned by using fluoride-free cleaning paste (Proxyt, fluoride-free prophy paste, Ivoclar Vivadent), rinsed, and then air-dried. Subsequently, the teeth were etched with 35% phosphoric acid for 15 seconds (UltraEtch, Ultradent), and after rinsing and air drying, a light curing adhesive was scrubbed into the preparation surface for 20 seconds and then dispersed with compressed air until an immobile film layer results (Adhese Universal VivaPen, Ivoclar Vivadent). Polymerization was performed for 10 seconds (1,400 mW/cm ^2^ ). The light-cured composite cement in neutral shade (Variolink Esthetic LC neutral, Ivoclar Vivadent) was applied onto prepared internal surface of each ceramic veneer that were gently seated with finger pressure. The excess cement was polymerized for 2 seconds and then removed with a scaler. Immediately after excess removal, the restoration margins were covered with glycerine gel (Liquid Strip, Ivoclar Vivadent) and polymerized from the facial, lingual and incisal aspects for 10 seconds each (light intensity of 1,400 mW/cm, Valo Cordless, Ultradent). After polymerization, the retraction cord and excess polymerized cement was removed. Finally, the margins were finished with carbide bur (Edenta AG) and polished with rubber points (Kenda) ( Figs. 5 , 6 and 7 ). The follow-up was performed 1 month after cementation and then annually.

### All-Ceramic Full Crowns

**Fig. 8:**
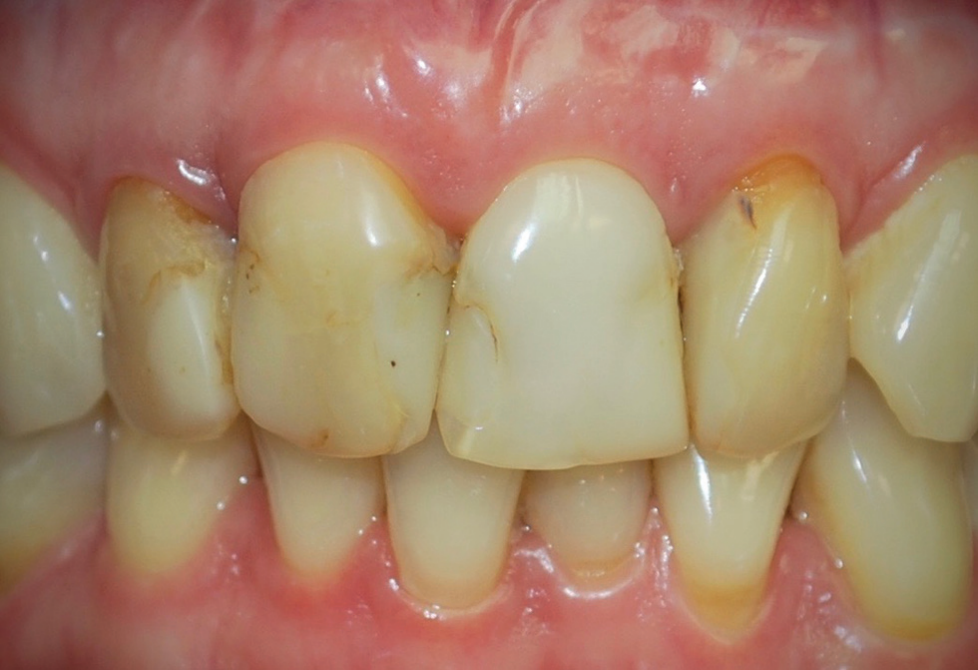
Intraoral frontal view of the anterior dentition. Note the unsatisfactory composite resin fillings, dentin exposures on teeth 12 and 22 and discolored teeth due to root canal treatment.

**Fig. 9:**
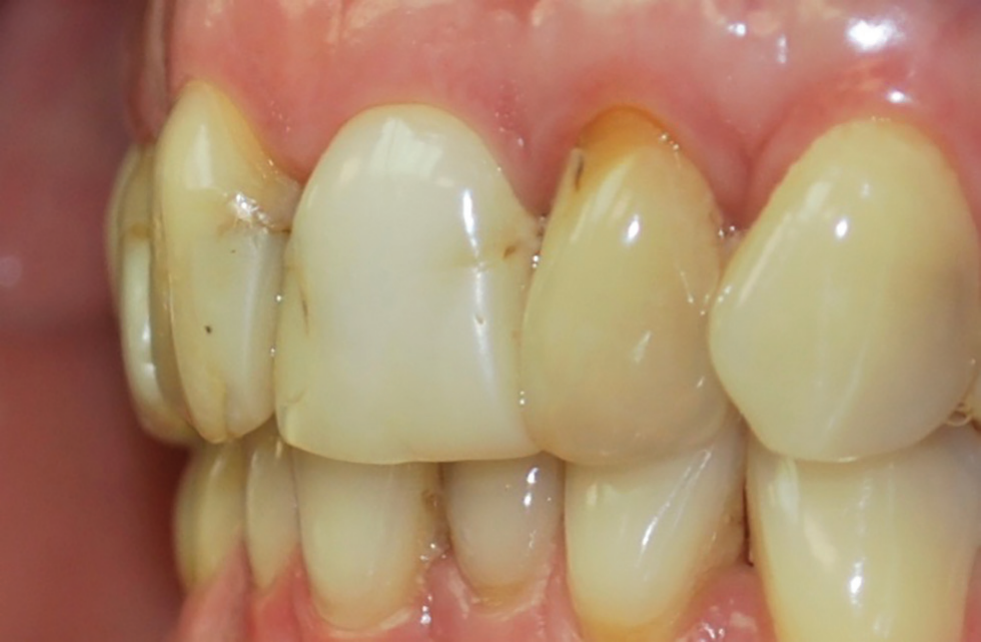
Intraoral lateral view of the anterior dentition. Note the teeth misalignments with length discrepancies.

The 40-year-old female patient sought treatment complaining about the esthetic of the maxillary anterior teeth ( Fig. 8 and 9 ). The patient’s chief complaint was unattractive smile. The extra-oral and intra-oral clinical examinations were performed and revealed half Angle Class III malocclusion right side and half Angle Class II malocclusion left side, good oral hygiene, unsatisfactory composite resin fillings (composite overhangs), discolored teeth due to root canal treatment (12, 11, and 22), and teeth misalignments with length disharmony between the central and lateral incisors. No periodontal problems or carious lesions were found. Radiographic examination revealed extended composite restorations of the upper incisors, endodontically treated teeth number 12, 11, and 22 with no findings of carious lesions or periapical pathology. The quality of endodontic treatments was assessed with a favorable outcome. Evaluation of the patient’s medical history was insignificant. Based on examination (teeth discolorations, endodontically treated teeth, dentin exposures, teeth misalignments), the all-ceramic full crowns were planned to restore all maxillary incisors. The diagnostic wax-up model was fabricated to provides a three-dimensional view of the future restoration. Diagnostic wax-up model improve communication between the restorative team and the patient. The silicone index fabricated according to diagnostic wax-up allows the clinician to control the amount of tooth reduction during preparation.

The first phase involved the selection of the color shade using a IPS e.max Shade Guide (Ivoclar Vivadent). Subsequently, the digital photography was performed to provide diagnostic information to the restorative team.

**Fig. 10:**
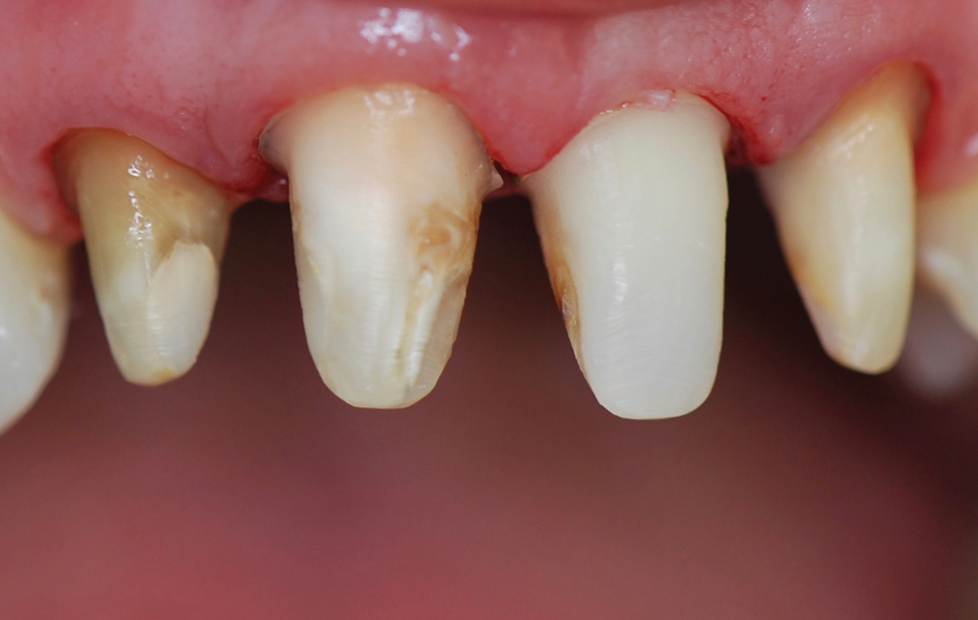
Prepared upper incisors.

The crown preparation involved the use of a round-end diamond bur (Edenta AG) to create three facial reduction grooves respecting the axial inclinations of the tooth. The bur was positioned on the facial cervical area so that the reduction would end at half of the bur’s diameter. The grooves were subsequently evened by using the cylindric diamond bur (Edenta AG). The gingival margin was prepared to 1.5 mm chamfer and was positioned 0.5 mm subgingivally. The depth of reduction was controlled by using the silicone index fabricated according to a diagnostic wax-up. Subsequently, the 3-mm incisal reduction was carried out. The next phase consisted of the interproximal and palatal wraparound. A thin tapered diamond bur was used to create an interdental space for the application of a larger diamond bur for the wraparound (Edenta AG). Subsequently, a round-end and ovoid diamond burs were used for the palatal reduction (Edenta AG). Extra-fine finishing diamonds were used to eliminate sharp angles and obtain smooth contours ( Fig. 10 ).

The next phase involved the impression at the same appointment, using addition silicone (Variotime, Kulzer) and a double-cord technique for gingival deflection. Compression cord with a small diameter was placed at the bottom of the sulcus (7 Siltrax AS, Pascal). Next, a more superficial thicker deflection cord was inserted in the entrance of the sulcus (1 Ultrapak, Ultradent). Gingival deflection was carried out for 5 minutes to allow the deflection cord to expand by water sorption. Before the impression was taken, the deflection cord was removed to obtain deflected sulcus, which allows penetration of the light-body impression material into the sulcus, beyond the preparation margins. Provisional restoration was created chairside with self-curing acrylic resin-based provisional restoration material (Structure 2SC, Voco).

In the laboratory, the lithium disilicate ceramic (IPS e.max Press A1 LT, Ivoclar Vivadent) was used for the fabrication of the frameworks, which were consequently veneered by using the layering technique (IPS e.max Ceram). The time between the final impression and cementation was 10 days.

**Fig. 11:**
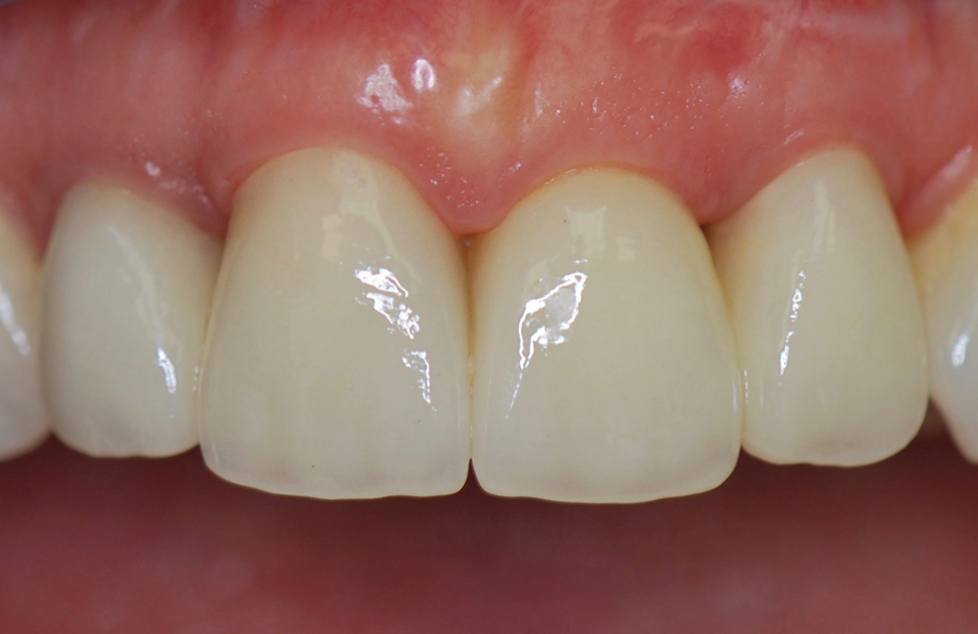
Postoperative frontal view 1 month after definitive placement of the crowns (IPS e.max Press, LT- framework, IPS e.max Ceram-veneering ceramic).

**Fig. 12:**
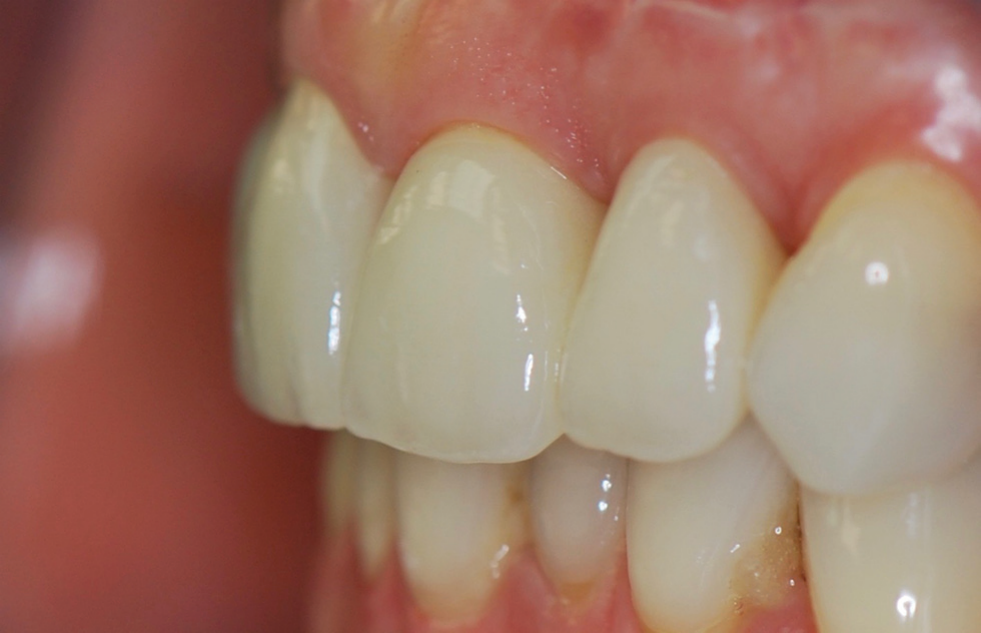
Postoperative lateral view 1 month after definitive placement of the crowns.

**Fig. 13:**
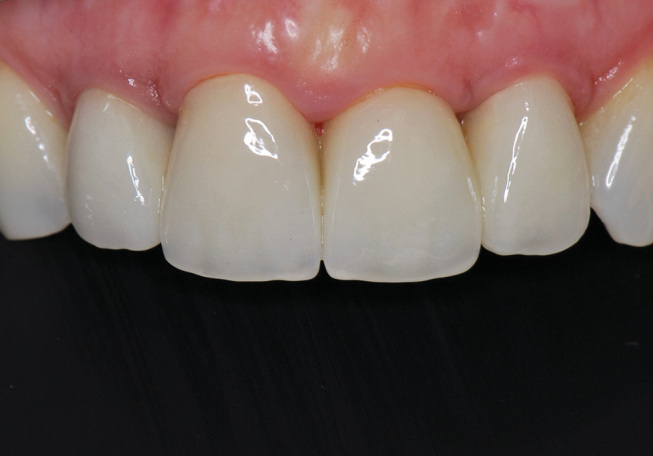
Two-year follow up of ceramic crowns.

Except for adhesive (Multilink Primer, Ivoclar Vivadent) and luting system (Multilink Automix transparent, Ivoclar Vivadent) used for the cementation of the crowns, the next clinical steps (try-in, surface treatment, finishing and polishing) were performed similarly as described in the previously presented case report. The follow-up was performed 1 month after cementation and then annually ( Figs. 11 , 12 and 13 ).

## Discussion

Silica-based all-ceramics have been proven effective in numerous clinical studies as an appropriate material for esthetic single tooth restorations. 17 23 24 25 Ceramic veneers are considered advantageous for maintaining tooth vitality and preserving hard tissues. 26

Full crown preparations require removal of 63 to 72% of tooth structure, while veneers require removal of only 3 to 30% of tooth structure. 27

On the other hand, the patient-related factors as well as amount and quality of remaining tooth tissue should be precisely evaluated when choosing between all-ceramic crowns and extended veneers. 19

The veneer preparation should be confined primarily within the enamel or should display a substantial (50–70%) enamel area, especially at the preparation margins.

Debonding of ceramic veneers has been reported when dentin comprises 80% or more of the tooth substrate. In contrast, debonding is highly unlikely when the preparation margins are placed in enamel. 19 28 29

Therefore, the longevity of all-ceramic restorations can be compromised in elderly patients because of enamel thickness, which diminishes over time. Especially, cervical area of the tooth may have little or no enamel. Moreover, there may be an increased load due to the lack of posterior dentition as well as risk of the microleakage incidence related to root dentin exposure. 19 28

Further, the condition of the tooth in terms of whether the tooth is vital or endodontically treated should be taken into consideration. Meijering et al demonstrated that veneers on nonvital teeth show higher risk to fail than veneers placed on vital teeth. 30 Another long-term study by Beier et al also demonstrated that veneers on nonvital teeth showed a significantly higher failure risk. 31 In contrast, von Stein-Lausnitz et al indicated that in endodontically treated maxillary central incisors with Class III defects, less invasive veneers appear to be more beneficial than ceramic crown restorations. 32

The presence of tooth discoloration is common for nonvital teeth. Due to the thinness, the masking ability of the ceramic veneers is limited. 33 Therefore, more reduction of the hard tissues may lead to better esthetic result of the full-crown restorations. When the ceramic veneers and full-crowns are used simultaneously in rehabilitation of anterior teeth, the value mismatch could be evident because of different ceramic thickness. Therefore, if discolored abutment tooth is presented, all other teeth should be restored with the same system to achieve a harmonic esthetic outcome. 19

## Conclusion

These case reports demonstrated two types of fixed restorations of the anterior dentition-extended ceramic veneers and full-coverage crowns.

When selecting an appropriate treatment, ceramic veneers should only be chosen when bonding is a completely feasible option. In the cases, when this attribute cannot be achieved (e.g., reduced enamel area-extensive composite restorations or dentin exposures, especially at the preparation margins; highly discolored teeth, when large amounts of enamel must be prepared to obtain the sufficient thickness and masking ability of the ceramic material; significant teeth misalignments) all-ceramic crowns seems to be the better treatment option.
